# Patient-Specific Fetal Dose Determination for Multi-Target Gamma Knife Radiosurgery: Computational Model and Case Report

**DOI:** 10.7759/cureus.1527

**Published:** 2017-07-31

**Authors:** Anna K Paulsson, Steve Braunstein, Justin Phillips, Philip V Theodosopoulos, Michael McDermott, Patricia K Sneed, Lijun Ma

**Affiliations:** 1 Radiation Oncology, University of California, San Francisco; 2 Department of Neurological Surgery, University of California, San Francisco

**Keywords:** gamma knife radiosurgery, cancer in pregnancy, brain metastases, fetal radiation dose

## Abstract

A 42-year-old woman at 29 weeks gestation via in vitro fertilization who presented with eight metastatic brain lesions received Gamma Knife stereotactic radiosurgery (GKSRS) at our institution. In this study, we report our clinical experience and a general procedure of determining the fetal dose from patient-specific treatment plans and we describe quality assurance measurements to guide the safe practice of multi-target GKSRS of pregnant patients. To estimate fetal dose pre-treatment, peripheral dose-to-focal dose ratios (PFRs) were measured in a phantom at the distance approximating the fundus of uterus. Post-treatment, fetal dose was calculated from the actual patient treatment plan. Quality assurance measurements were carried out via the extrapolation dosimetry method in a head phantom at increasing distances along the longitudinal axis. The measurements were then empirically fitted and the fetal dose was extracted from the curve. The computed and measured fetal dose values were compared with each other and associated radiation risk was estimated. Based on low estimated fetal dose from preliminary phantom measurements, the patient was accepted for GKSRS. Eight brain metastases were treated with prescription doses of 15-19 Gy over 143 min involving all collimator sizes as well as composite sector mixed shots. Direct fetal dose computation based on the actual patient’s treatment plan estimated a maximum fetal dose of 0.253 cGy, which was in agreement with surface dose measurements at the level of the patient’s uterine fundus during the actual treatment. Later phantom measurements also estimated fetal dose to be in the range of 0.21-0.28 cGy (dose extrapolation curve R^2 ^= 0.998). Using the National Council on Radiation Protection and Measurements (NCRP) population-based model, we estimate the fetal risk of secondary malignancy, which is the primary toxicity after 25 weeks gestation, to be less than 0.01%. Of note, the patient delivered the baby via scheduled cesarean section at 36 weeks without complications attributable to the GKSRS procedure. GKSRS of multiple brain metastases was demonstrated to be safe and feasible during pregnancy. The applicability of a general patient-specific fetal dose determination method was also demonstrated for the first time for such a treatment.

## Introduction

Gamma Knife stereotactic radiosurgery (GKSRS) has been widely adopted as a definitive or adjuvant treatment of various intracranial lesions, including multiple brain metastases [1-7]. It is rare for a pregnant woman to present with brain metastases and be referred for the GKSRS procedure. For some patients early delivery may be an option to facilitate more comprehensive oncologic imaging and management, however, the risks and benefits must be carefully assessed in a multi-disciplinary setting. One of the key concerns for the procedure is possible radiation toxicity to the fetus, which is dependent on the gestational age at the time of radiation exposure. In the first trimester during organogenesis, the major risks are teratogenicity and growth retardation, in the second trimester risk of growth retardation increases and there is a possible reduction in IQ with radiation exposure, and in the third trimester the primary risk is radiation-induced malignancy (leukemia) in the developing fetus [2]. There is limited data on the use of GKSRS for the treatment of brain metastases in pregnant patients and most studies describe managing patients on the 201-source configuration of the early GKSRS systems [1, 4].

With the introduction of Gamma Knife Perfexion (PFX) system, a single universal self-shielded tungsten collimator was employed [8-9], which significantly reduced the distal peripheral dose and external radiation exposures. The external radiation exposure is low enough such that some centers have considered installing a glass window in the treatment vault [8]. In light of enhanced technical capability and the low external radiation dose of the PFX system [10-11], we accommodated treatment of a rare GKSRS case at our institution, treating a pregnant patient with eight brain metastases of varying size throughout the cerebellum and cerebrum. Prior to treatment delivery, we endeavored to determine a reliable method to estimate the patient-specific fetal dose, and whether it is practical and safe to treat pregnant patients in general with multiple brain tumors with Gamma Knife Perfexion. To answer these questions, we developed an empirical computation method and concurrently performed in-phantom dose measurements to cross check the dose calculation results. One major goal of the study was to validate the technical suitability and safety of GKSRS in managing pregnant patients with complex and/or multiple brain lesions.

In the following sections, we first describe the clinical and technical experience of treating the pregnant patient. Then we describe a general dose computation method applicable to fetal dose determination and associated quality assurance measurement procedures and results. The study also details the approach for determining composite dose exposure to distal critical organs (e.g., fetus, heart/pacemaker, thyroid, etc.) for patient-specific GKSRS peripheral radiation exposure risk assessment, especially for multiple targets.

## Technical report

Case description

A 42-year-old gravida 1, para 0 female status post in vitro fertilization, with prior history of breast cancer presented for treatment of brain metastases in December 2014. Her primary cancer was BRCA1 positive, triple negative pT3N0M0, AJCC stage IIB, poorly differentiated, multifocal, grade 3 infiltrating ductal carcinoma of the right breast diagnosed in March 2012. She had previously received six cycles of neoadjuvant iniparib, cisplatin and gemcitabine with a near complete response to treatment. She subsequently had bilateral skin sparing mastectomies followed by post-operative chest wall and axillary nodal radiation, completed in early 2013. She underwent bilateral breast reconstruction eight months later.

Approximately one year after completion of her treatment she presented to an outside hospital with progressive right arm and right leg weakness. Non-contrast magnetic resonance imaging (MRI) showed a large left frontal cystic mass with surrounding edema and mass effect with 2 mm of left-to-right midline shift. Surgical planning MRI two days later showed at least three T1 hypointense, T2/fluid-attenuated inversion recovery (FLAIR) hyperintense lesions including the 4.9 x 3.6 x 4.2 cm high posterior left frontal dominant lesion, a 1.7 cm cerebellar mass with surrounding edema, and a 7 mm partially cystic mass in the right anterior temporal lobe. Additional lesions could not be excluded due to lack of intravenous contrast. She had a gross total resection of the large left frontal mass at 24 weeks gestation with pathology confirming metastatic triple negative breast cancer. The patient was referred for Gamma Knife radiosurgery one month post-operatively. After the fetal dose estimation and risk assessments as described in the following sections, the patient received GKSRS treatment as a routine procedure due to the estimated low risk of fetal radiation exposure. During treatment, the surface radiation dose was measured at the level of the uterine fundus using MOSFET dosimeters (Best Medical, Ottawa, ON, Canada) to compare with calculated estimates and phantom measurements.

Calculated fetal dose

First, we measured the generic peripheral dose-to-focal dose ratios (PFRs) of individual collimator sizes (16 mm, 8 mm, 4 mm and 0 mm, where 0 mm denotes blocked isocenter for the purpose of computing composite transit dose between isocenters). PFR is here defined as

                                       PFR (s,d) = PD (s,d)/FD (s,d_0_)  (1)

where PD (s,d) is the distal peripheral dose rate at a distance of d for the collimator size s and FD (s,d_0_) is the dose rate of a given collimator at the focal point (d_0_) corresponding to the Leksell G-frame coordinates of (x, y, z) = (100, 100, 100) mm. Once the PFRs were known, the patient-specific fetal dose (D_f_) was computed as follows:

                                        D_f _= D_0_ t_b_w_b _PFR_b _+ ∑_(i=1)_^N ^d_i_t_i _(w_i,16_ PFR_16 _+ w_i,8_PFR_8 _+ w_i,4_ PFR_4 _+ w_i,b_ PFR_b_) (2)

where N is the total number of isocenters for the treatment plan, D_0_ is the reference dose rate, d_i_ and t_i_ are the central dose rate and irradiation time for the i^th^ shot, both of which values are provided in the treatment plan printout under the shot details section, t­_b_ ­­is the composite shot transit time including shuttle time between shots, (i.e., t­_b_ = total treatment time – total beam-on time), (PFR­_b_, PFR­_4_, PFR­_8_, PFR­_16_) are the PRF values for 0-mm, 4-mm, 8-mm and 16-mm collimator, respectively, and {w_­i,b_, w_­i,4_, w­_i,8_, w_­i,16_} are relative sector weighting factor for the i^th^ shot, i.e., w_i,b_ = n_i,b_/8 w_i,4_ = n_i,4_/8; w_i,8_ = n_i,8_/8; w_i,16_ = n_i,16_/8, where n_i,4_ , n_i,8_ , n_i,16_ are the number sectors open for the i^th^ shot, respective. For example, if the 8-sector opening for a composite i^th^ shot is given {b, 4, 4, 8, 8, 8, 16, 16}, then it follows that {w_b,i_ = 1/8 = 12.5%, w_4,i_ = 2/8 = 25%, w_8,i_ = 3/8 = 37.5%, w_16,i_ = 2/8 = 25%}. Of note, the current GKSRS treatment planning system (LGP version 10.2) assumed D_b_, the background dose rate for the blocked shot, to be 0.00 cGy/min.

Phantom measurements

**Figure 1:**
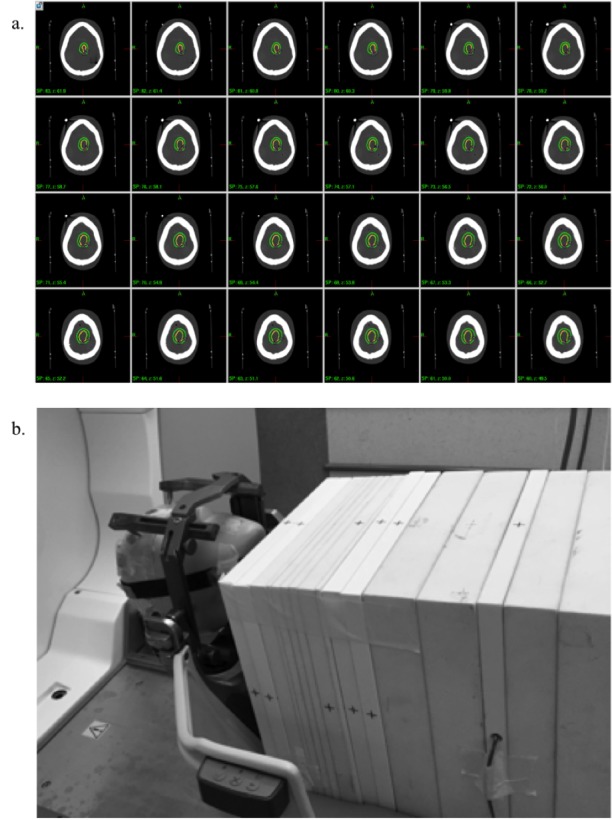
Projected treatment plan and experimental set-up. Illustration of the patient’s projected treatment plan on the computed tomography (CT) study of a head phantom (a and b) shows the experimental set up for the ionization chamber measurements.

To directly verify and cross check the computed value of Equation 2, direct dose measurements were carried out using computed tomography (CT) based planning (Figure 1a). The experimental setup for the measurements is illustrated in Figure 1b, where measurements were carried out using a compact ionization chamber (Wellhofer-CC13, IBA Dosimetry, Bartlett, TN) placed in a stack of solid water blocks and coupled with a humanoid head phantom (CyberKnife, Sunnyvale, CA) attached to the Leksell G-frame (Elekta, Atlanta GA).

The head phantom served as patient head simulation and solid water blocks approximated the dose scattering condition (Figure 1b). Thin-cut CT scans (1.5 mm) of the phantom were also obtained and actual patient treatment plan was projected on the phantom to simulate the actual treatment (Figure 1a). For each projected treatment plan, ionization chamber measurements were carried out by systematically shifting the chamber inferiorly inside a solid water block. Similarly, the PFR was measured by placing single shots (i.e., 16 mm, 8 mm, 4 mm and 0 mm) at the center of head phantom and taking measurements at locations approximating the patient’s uterine fundus, which was obtained by tape measure measurement during the time of initial patient consultation. Finally, the distal peripheral dose at any distance including the fetal locations was obtained by empirically fitting the measurement data. The results from the curve were compared with the direct computation result of Equation 2.

Patient treatment planning and treatment

**Figure 2:**
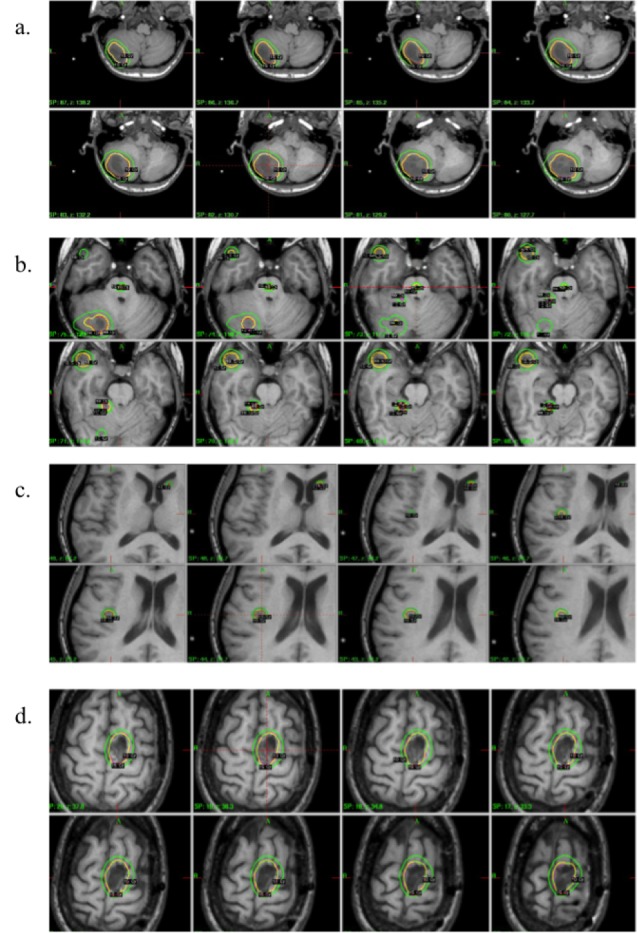
Axial MR slices for target delineation. Axial MR slices for the pregnant patient depicting the target locations (N = 8) and associated the isodose distributions. Note the small lesion inside the brainstem (2b), the large cerebellar metastasis (2a) and frontal lobe resection cavity (2d). The yellow lines show the prescription dose contours and the green lines show the 10-Gy contour line.

At the time of radiosurgery, the patient was 29 weeks pregnant, in her third trimester. The Gamma Knife planning MRI (Figure 2) was performed without gadolinium contrast, which is contraindicated during pregnancy, included T1 3D fast spoiled gradient echo (FSPGR), 3D fast spin echo (FSE) T2, increased signal intensity (ISI), and T2/FLAIR sequences. With guidance from neuroradiology, a total of eight lesions, including the left frontal resection cavity, were identified for treatment on the T2 FLAIR sequences. Axial isodose contours for the outlined targets are shown in Figures 2a-2d. The mean target volume was 2.83 ± 4.7 cc (range, 0.03-12.4 cc) and the targets were treated with prescription doses of 15-19 Gy based upon size and location. No margin for target uncertainty was added to the GTV, however, five of the eight targets were prescribed to isodose lines higher than 50% (range, 67-87%). The total target volume was 22.6 cc. The treatment plan used 4, 8 and 16 mm collimation and a total of 32 distinct isocenters. The total in-room treatment time was 143 min with a total beam-on time of 138 minutes. The surface dose measurement at the level of the uterine fundus for the entire treatment was <0.05 cGy.

Of note, the patient delivered the baby via scheduled caesarian section at 36 weeks without complications attributable to the GKSRS procedure. At her two-month follow-up MRI with gadolinium contrast, there was significant decrease in five of the treated metastases, two areas of FLAIR hyperintensity which may represent treated metastases with resolution of enhancement, four new untreated metastases, and two lesions which in retrospect, were punctate dots of FLAIR abnormality at the time of her Gamma Knife planning MRI. At this point, the patient also had extra-cranial progression of the disease for which she was started on systemic chemotherapy. Since her initial Gamma Knife treatment, she has had three additional GKSRS procedures and whole brain radiotherapy. She remains alive 3.5 years after her initial presentation with intracranial disease and at last follow-up she had ECOG performance status of 1.

**Figure 3:**
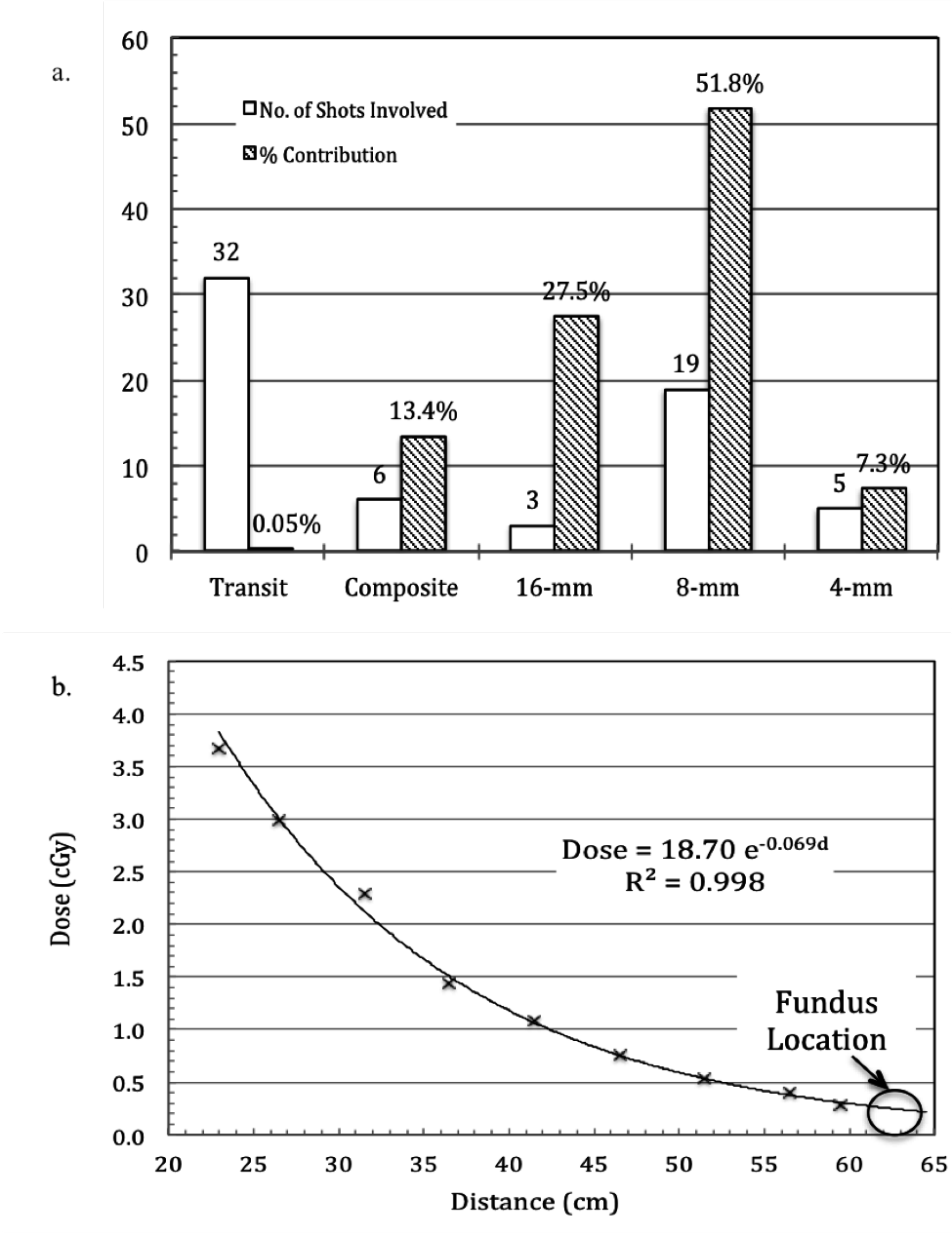
Individual shot dose contribution and dose extrapolation. Relative contributions from individual shots for the computed maximum fetal dose at the fundus of the uterus location based on the actual patient’s treatment planning (a) and the fitting results for the direct extrapolation dose measurements. Note the excellent fitting result yielding R2 = 0.998 with the final fitted formula shown.

The PFRs for the 16-mm, 8-mm, and 4-mm collimators were determined to be 0.0013 ± 0.0000, 0.0048 ± 0.0001, and 0.00038 ± 0.0003, respectively. Although the GKSRS treatment planning system assumed D_b _= 0.00 cGy/min, for more realistic estimate in the study we substituted the maximum in-phantom dose rate measurement when blocking all the sectors and applied PFR for the 0-mm for the calculation, i.e., PFR_b _= 0.0035 and D_b_ = 0.028 Gy/min. Substituting all the PFR values as well as the focal dose rate from the patient’s treatment plan, the maximum fetal dose at the location of uterus fundus was calculated to be 0.253 cGy, which was in agreement with surface dose measurements using MOSFETs at the level of the patient’s uterine fundus during the actual treatment. The robustness of the calculated value was further confirmed by varying all the PFR values by 3s or ~40% and the maximum fetal dose detected was found to be <0.35 cGy for all the cases. Figure 3a plots the individual collimator/combination contributions and the number of shots associated with such a calculation. As shown in Figure 3a, 51.8% of the calculated dose was from the 19.8-mm shots, whereas the three 16-mm shots contributed 27.5%. The total shot transit time only contributed approximately 0.005% of the total dose.

The extrapolation dose measurement result is plotted in Figure 3b, where nearly perfect exponential fit was obtained, i.e., R2 = 0.998 from linear regression in logarithmic scaling of both x- and y-axis. From the fitted curve, the maximum fetal dose near the fundus of the uterus was estimated to be 0.278 to 0.211 cGy, which agreed well with the calculated value of 0.253 cGy from the patient’s treatment planning data. Noting excellent fitting result of Figure 3b, the dose to other critical structure of the patient was also obtainable. For example, the dose to the thyroid was found to be <2.5 cGy, the maximum heart dose was <1.5 cGy, etc.

## Discussion

GKSRS of a pregnant patient with multiple brain metastases (N = 8) is detailed in this report. Compared with previous Gamma Knife systems (Models U-4C) for which distal peripheral dose was reported to be on the order of 10 cGy for a simple treatment [1], distal peripheral dose was found to be 1-2 orders of magnitude lower for the PFX system, as in the current study. A patient-specific fetal dose computation procedure and extrapolation dosimetry measurements were developed and successfully carried out for the patient treatment.

Good correlation was found between calculations and measurements. The maximum fetal dose for this case was determined to be <0.3 cGy. Based on the current National Council on Radiation Protection and Measurements (NCRP) recommendation of 0.5 cGy whole-fetus dose limit for the general public [12], the treatment for the patient was deemed safe. A conservative estimate of the probability of toxicity, in this case radiation-induced malignancy, was <0.002% using NCRP population-based risk model (i.e., 0.006%/cGy to the whole fetus).

It is evident that the measurement and calculation method presented are readily translatable to other distal locations given that patient-specific measurements are performed, as demonstrated in Equation 2.

## Conclusions

In summary, the results of this study have demonstrated that GKSRS is a technically feasible modality and safe by a conservative standard for treatment of patients with multiple brain metastases during the 2nd-3rd trimesters of pregnancy. Special care must be taken by clinicians to ensure the dose to the fetus is reliably estimated via both the patient-specific treatment plan and in-phantom measurements as demonstrated by this study.
